# Development and validation of a predictive model for postoperative deep vein thrombosis in elderly patients undergoing hip fracture surgery: A retrospective study

**DOI:** 10.1097/MD.0000000000049000

**Published:** 2026-05-22

**Authors:** Zheng Xu, Jie Liu, Weiting Wu, Xing Liu, Xuli Yang

**Affiliations:** a School of Public Health, Jiangxi Provincial Key Laboratory of Disease Prevention and Public Health, The First Affiliated Hospital, Jiangxi Medical College, Nanchang University, Nanchang, Jiangxi, China; d Department of Medical Equipment, The First Affiliated Hospital, Jiangxi Medical College, Nanchang University, Nanchang, Jiangxi, China; e Department of Medical Service, The First Affiliated Hospital, Jiangxi Medical College, Nanchang University, Nanchang, Jiangxi, China; b Scientific Research Administration Office, Jiangxi Provincial Center for Disease Control and Prevention, Nanchang, Jiangxi, China; c Jiangxi Provincial Key Laboratory of Major Epidemics Prevention and Control, Nanchang, Jiangxi, China

**Keywords:** deep vein thrombosis, hip arthroplasty, nomogram model

## Abstract

This study aimed to systematically examine the incidence of deep vein thrombosis (DVT) after hip surgery and identify its key determinants. The findings are intended to inform the refinement of postoperative risk assessment tools, the optimization of prevention strategies, and the development of personalized nursing protocols. This retrospective cohort study consecutively enrolled elderly patients aged ≥ 60 years who underwent hip fracture surgery at the First Affiliated Hospital of Nanchang University between January and September 2024 and had complete medical records. The primary outcome was postoperative DVT confirmed by imaging. Predictive factors were identified through univariate and multivariate logistic regression analyses, and a nomogram prediction model was constructed. A total of 710 patients were included, with a postoperative DVT incidence of 12.11% (86/710). Significant differences were observed between the DVT group and the non-DVT group in terms of 24-hour post-admission assessment and venous thromboembolism scores (*P* < .05).Multivariate logistic regression revealed that a specialized risk score within 24 hours of admission (OR = 4.326, 95% CI: 2.00–9.35, *P* < .001), elevated preoperative C-reactive protein levels (OR = 1.018, 95% CI: 1.01–1.03, *P* < .05), perioperative blood transfusion (OR = 0.212, 95% CI: 0.10–0.43, *P* < .001), Postoperative graded elevation in D-dimer levels (OR = 2.691, 95% CI: 1.08–6.37, *P* < .05), and preexisting DVT risk scores (OR = 1.711, 95% CI: 1.35–2.17, *P* < .001) were identified as independent predictors of postoperative DVT in elderly patients undergoing hip replacement surgery. The constructed nomogram model demonstrated excellent predictive performance (concordance index: 0.873). In elderly patients receiving hip replacement surgery, 5 independent predictors of postoperative DVT were identified. These factors were integrated into a bar-chart-based risk assessment tool, which quantifies individualized thrombotic risk through a visual scoring system. This model provides clinicians with a practical method for early risk stratification, supporting targeted prevention strategies and postoperative monitoring in routine care.

## 1. Introduction

Older adults are more susceptible to femoral head necrosis and fragility fractures of the femoral neck due to hip osteoarthritis and osteoporosis. Total hip arthroplasty (THA) is the most effective treatment for these conditions. However, the occurrence of deep vein thrombosis (DVT) postoperatively significantly compromises patient outcomes, leading to prolonged hospital stays and increased healthcare costs. Progression to pulmonary embolism may result in perioperative mortality.

Despite being a key quality control indicator in perioperative management, postoperative DVT remains a significant complication in elderly patients undergoing hip surgery. Epidemiological studies indicate that the incidence of DVT in Asian populations can reach as high as 41.9%. Even with standard prophylactic measures in place, the incidence rate among high-risk populations persists between 11.1% and 29.4%,^[1]^ due to factors such as advanced age and the inherent trauma of the surgical procedure itself,^[2,3]^ Existing research has confirmed that advanced age, surgical trauma, and postoperative immobilization are all independent risk factors for DVT.^[4]^ However, in the specific population undergoing hip surgery in the elderly, these factors may interact complexly with the patient’s underlying condition (such as the severity of osteoporosis, extent of bone necrosis, and comorbidities), collectively influencing the individual’s overall risk of thrombosis.However, current clinical risk assessment models are predominantly derived from Western population data, Their applicability and predictive accuracy in elderly Asian patients undergoing hip surgery remain questionable, as these tools often fail to adequately integrate the combined effects of procedure-specific and regional risk factors.^[5]^ This limitation underscores a critical gap in current risk stratification, highlighting the need for a more personalized and population-specific predictive tool. Therefore, this study aims to develop and validate a nomogram model for predicting postoperative DVT in elderly patients undergoing hip surgery. We hypothesized that integrating key preoperative and perioperative clinical indicators would yield a predictive model with good discrimination and calibration. The specific objectives were to: identify independent predictors of postoperative DVT using retrospective clinical data; construct a visual nomogram prediction model based on multivariate logistic regression analysis; and evaluate the model’s predictive performance through internal validation.

## 2. Objects and methods

### 2.1. Research subjects

This single-center retrospective study was approved by the Ethics Committee of the First Affiliated Hospital of Nanchang University, which granted a waiver of informed consent due to the retrospective design.

Inclusion criteria: Age ≥ 60 years, regardless of sex; No prior history of venous thrombosis before the current hospitalization; Underwent THA during the hospitalization period; and Complete clinical records available, including preoperative, intraoperative, and postoperative data. Exclusion criteria: DVT confirmed by imaging prior to surgery; Incomplete or missing key medical records; Severe hepatic, renal, or cardiac dysfunction, acquired immunodeficiency syndrome, malignant tumors, or other critical illnesses; Pathological hip fractures secondary to primary or metastatic tumors; Transfer to another hospital or department during the study period, resulting in unavailability of postoperative outcome data; Requirement for long-term therapeutic anticoagulation for indications other than DVT prevention; and Lack of documented postoperative follow-up data for at least 30 days.

### 2.2. Research data sources

A retrospective review of the hospital’s electronic medical record system was conducted. Patients who underwent THA were screened, and relevant clinical and follow-up data were extracted. This specifically included:

1. General information: age, gender, medical history, etc;
2. Clinical data: length of hospital stay, surgery duration, postoperative complications, etc;
3. Laboratory indicators: Pre- and postoperative white blood cell count, red blood cell count, platelet count, hemoglobin, neutrophil percentage, C-reactive protein (CRP), D-dimer, etc;
4. Nursing risk assessment: Scores from scales measuring functional independence, pain, pressure ulcers, falls, and venous thromboembolism.

Additionally, patient contact information was collected for follow-up. Anticoagulant medication use was documented, and adverse events such as bleeding and recurrent thrombosis were monitored to evaluate treatment efficacy and recovery status.

### 2.3. DVT follow-up and outcomes

#### 2.3.1. Patient follow-up

Follow-up protocol: All patients underwent a 3-month postoperative follow-up period starting from the day of surgery. During this period, recovery status and risk of DVT were systematically assessed through a combination of outpatient follow-up visits and telephone follow-ups. Key follow-up points included outpatient visits at 1 month (30 ± 7 days) and 3 months (90 ± 7 days) postoperatively, supplemented by necessary telephone follow-ups to monitor patient status. Follow-up content covered symptoms, signs, functional recovery, anticoagulation therapy adherence, and any adverse events in the affected limb.

Outcome definition: The primary outcome of this study is symptomatic or asymptomatic DVT of the lower extremities occurring during follow-up. DVT diagnosis strictly adheres to the “Guidelines for Diagnosis and Treatment of Deep Vein Thrombosis (Third Edition).” Confirmation requires detection of thrombus formation within deep veins via color Doppler ultrasound at any postoperative time point, followed by verification by a vascular surgeon.

#### 2.3.2. Diagnostic criteria for DVT

According to the Guidelines for Diagnosis and Treatment of Deep Vein Thrombosis (Third Edition), the diagnostic criteria for lower extremity DVT are as follows: Swelling and pain in the lower extremity, accompanied by painful cramps and abnormal gait, which may be relieved by bed rest or elevation of the affected limb; Purplish-red or cyanotic skin on the lower extremity with elevated skin temperature and dilated superficial veins, accompanied by possible fever, tachycardia, and elevated white blood cell count, though body temperature remains below 38.5°C; Positive Homans sign on straight leg raise test, presence of pitting edema in the anterior tibial region, and tenderness in the quadriceps and gastrocnemius muscles; and Color Doppler ultrasound reveals deep vein obstruction. A diagnosis of lower extremity DVT is confirmed when at least one of criteria and criterion are present. Ultrasound examination is recommended as a routine test for all patients to assess for postoperative DVT.

### 2.4. Quality control

Develop a standardized protocol and train research personnel; For at least 10% of the randomized sample, conduct independent double data entry by 2 data entry clerks. Compare discrepancies, trace back to source records for correction, and calculate data entry consistency rates; Research coordinators or quality managers not directly involved in follow-up shall periodically (e.g., monthly) review completed follow-up case data to verify completeness, timeliness, and accuracy, generating monitoring reports; and The entire study process shall adhere to ethical standards and undergo regular review.

## 3. Statistical analysis

Data analysis was performed using SPSS 26.0, and line charts were generated using the R (version 4.4.2) programming language.

Continuous variables were tested for normality using the Kolmogorov–Smirnov test. Normally distributed data were presented as mean ± standard deviation and compared using independent samples *t*-tests; non-normally distributed data were presented as median (interquartile range) and compared using Mann–Whitney *U* tests. Categorical variables were presented as frequencies (percentages) and compared using chi-square tests or Fisher exact tests, as appropriate. For comparisons involving multiple categorical variables (e.g., American Society of Anesthesiologists classification), omnibus tests were performed first, followed by pairwise comparisons with Bonferroni correction to adjust for multiple testing.

Candidate predictors were selected based on 2 aspects: clinical relevance, informed by prior evidence from orthopedic and thrombosis literature regarding factors potentially associated with postoperative DVT in elderly hip surgery patients; and data availability within the retrospective dataset. Initially included variables encompassed demographic characteristics (e.g., age, sex), preoperative laboratory indicators (e.g., CRP, D-dimer), surgery-related factors (e.g., surgical approach, blood transfusion status), and clinical risk assessment scores.

The cohort was randomly divided into training and internal validation sets in a 7:3 ratio. Baseline characteristics were compared between the 2 sets to ensure comparability. In the training set, univariate analyses were conducted to compare all candidate variables between the DVT and non-DVT groups. Variables with *P* < .05 in univariate analysis were entered into a multivariable binary logistic regression model using forward stepwise selection. Prior to model construction, multicollinearity was assessed using variance inflation factors, with a threshold of >10 indicating potential collinearity. In the validation set, model performance was assessed in terms of discrimination (area under the receiver operating characteristic curve) and calibration (Hosmer–Lemeshow test). Internal validation was performed using bootstrapping with 1000 resamples to calculate the optimism-corrected concordance index (C-index). Decision curve analysis (DCA) was conducted to quantify the clinical net benefit across a range of risk thresholds.^[6,7]^ A nomogram was constructed based on the final multivariable model. Statistical significance was set at *P* < .05 (two-tailed).

## 4. Results

### 4.1. Basic characteristics

**Figure 1.**
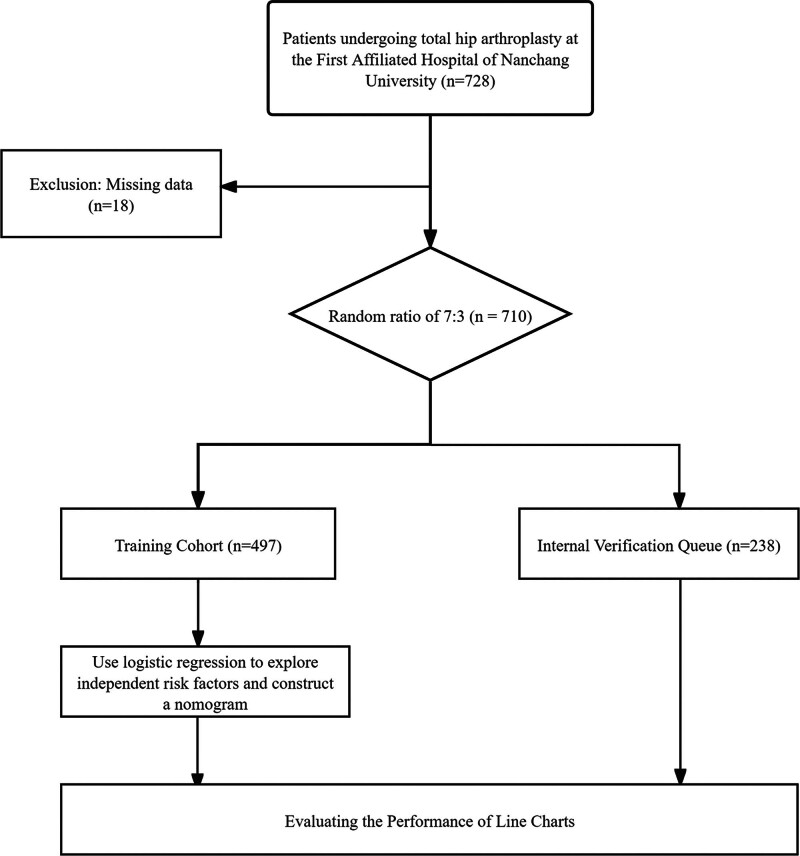
Patient screening flowchart.

This study enrolled 728 THA patients from the First Affiliated Hospital of Nanchang University, among these, 18 cases had missing data, and 710 cases were ultimately included (patient clinical characteristics: Table 1). The incidence rates of DVT in the training and internal validation cohorts were 11.8% (59/497) and 12.7% (27/213), respectively (Fig. 1).

**Table 1:** Clinical characteristics of patients [M (P25, P75)] OR [n (%)].

Indicator	No postoperative DVT events occurred (n = 438)	Postoperative deep vein thrombosis events (n = 59)	χ^2^ (*H*), *t*/Fisher exact/*U* test (*Z*)	*P*
Gender				
Male	174 (39.73)	18 (30.51)	0.172	>.05
Female	264 (60.27)	41 (69.49)		
Age (yr)				
60–74	237 (54.11)	21 (35.59)	8.068	<.001
75–89	179 (40.87)	32 (54.24)		
≥90	22 (5.02)	6 (10.17)		
BMI	22.24 ± 3.00	21.37 ± 3.57	4.777	<.05
Hypertension				
No	233 (53.20)	30 (50.85)	0.115	>.05
Yes	205 (46.80)	29 (49.15)		
Diabetes				
No	370 (84.47)	51 (86.44)	0.155	>.05
Yes	68 (15.53)	8 (13.56)		
Condition assessment within 24 h of admission				
1	420 (95.90)	46 (77.97)	24.282	<.001
2	16 (3.65)	8 (13.56)		
3	2 (0.45)	5 (8.47)		
Indications for total hip arthroplasty				
Primary or secondary hip osteoarthritis	232 (52.97)	49 (83.05)	19.167	<.001
Elderly patients with hip trauma fractures	170 (38.81)	8 (13.56)		
Avascular necrosis of the femoral head	36 (8.22)	2 (3.39)		
Preoperative DVT risk classification				
Low risk (1–2 points)	61 (16.35%)	5 (1.16%)	27.351	<.001
Moderate risk (3–4 points)	94 (21.15%)	5 (2.33%)		
High risk (5–6 points)	45 (9.29%)	4 (8.14%)		
Very high risk (7–8 points)	232 (52.24%)	36 (75.58%)		
Very high risk (≥9 points)	6 (0.97%)	9 (12.79%)		
Pressure ulcer risk assessment				
Very low risk	315 (75.33)	19 (53.49)	16.845	<.001
Low risk	97 (20.03)	5 (30.23)		
Moderate risk	15 (3.04)	5 (10.47)		
High risk	7 (1.60)	30 (4.65)		
Functional independence assessment score				
Minimal dependency	106 (24.20)	2 (3.39)	25.249	<.001
Moderate dependency	125 (28.54)	47 (79.66)		
Severe dependency	207 (47.26)	10 (16.95)		
Fall risk assessment				
Low risk	111 (25.34)	17 (28.81)	4.909	.086
Moderate risk	68 (15.53)	15 (25.43)		
High risk	259 (59.13)	27 (45.76)		
Affected side				
Right	226 (51.60)	25 (42.37)	1.961	.483
Left	212 (48.40)	34 (57.63)		
Blood transfusion				
Yes	40 (9.13)	22 (33.90)	37.752	<.001
No	398 (90.87)	37 (66.10)		
Preventive measures				
0	15 (3.37)	4 (6.80)	15.851	<.001
1	35 (8.17)	5 (8.43)		
2	3 (0.48)	4 (6.80)		
3	385 (87.98)	46 (77.97)		
Length of hospital stay	13.08 ± 4.90	17.54 ± 8.77	2.714	<.001
Surgery duration (h)	1.58 ± 0.60	1.54 ± 1.18	1.781	<.001
Intraoperative blood loss (mL)	235.46 ± 123.29	248.47 ± 218.78	2.624	<.001
VTE risk scor	5.63 ± 2.36	7.71 ± 1.24	6.703	<.001
Preoperative WBC count	6.19 ± 2.43	7.55 ± 2.85	1.256	.061
Preoperative CRP	1.65 ± 7.49	6.67 ± 7.21	2.740	<.001
Preoperative D-dimer	3.88 ± 0.66	3.38 ± 0.67	1.471	<.001
Red blood cell count	1.34 ± 0.55	1.11 ± 0.46	1.281	.028
Lymphocyte count	11.38 ± 0.31	12.16 ± 2.69	1.397	.034
Prothrombin time	30.14 ± 3.99	29.62 ± 4.32	0.945	.648
Activated partial thromboplastin time	3.55 ± 0.93	4.97 ± 8.69	1.209	.068
Fibrinogen	13.08 ± 4.90	17.54 ± 8.77	2.714	<.001
Postoperative D-dimer grading				
>0.5	244	51	20.357	<.001
≤0.5	194	8		

BMI = body mass index, CRP = C-reactive protein, DVT = deep vein thrombosis, VTE = venous thromboembolism, WBC = white blood cell.

### 4.2. Predictors of deep vein thrombosis following total hip arthroplasty

Univariate analysis revealed significant differences between the DVT group and the non-DVT group in multiple baseline characteristics (all *P* < .05). Patients in the DVT group were older (76.8 ± 8.5 years vs 73.0 ± 8.1 years), had more severe condition assessments at admission (proportion graded 2–3: 22.03% vs 4.11%), and exhibited more pronounced preoperative inflammation and hypercoagulability (CRP: 6.67 ± 7.21 mg/L vs 1.65 ± 7.49 mg/L; D-dimer: 3.38 ± 0.67 mg/L vs 3.88 ± 0.66 mg/L), and higher intraoperative transfusion rates (37.29% vs 9.13%), among others.The incidence of DVT in Level 1 patients within 24 hours of admission was significantly lower than in Level 2 (9.9% vs 33.3%) and Level 3 (9.9% vs 71.4%) patients. Indications: Patients with primary or secondary hip osteoarthritis exhibited a significantly higher venous thromboembolism (VTE) incidence than elderly patients with hip trauma fractures (17.4% vs 4.5%); Differences among other groups were not statistically significant. Preoperative DVT risk stratification assessment: VTE incidence increased with higher risk levels. The incidence in the very high-risk group (≥9 points) (60.0%) was significantly higher than in all other groups (adjusted *P* < .005). Pressure ulcer risk and functional independence: VTE incidence showed an upward trend with increasing risk or declining functional independence. Regarding functional independence, the incidence among non-independent patients (18.5%) was significantly higher than among fully independent (1.9%) and partially independent (7.4%) patients. Significant differences in VTE incidence were observed across groups receiving different numbers of preventive measures (overall *P* < .05). However, pairwise comparisons between groups failed to reach statistical significance after Bonferroni correction (corrected *P* > .0083).

Prior to multivariate analysis, collinearity diagnostics were performed, with all results <5, indicating no collinearity. Variables significant in univariate analysis were included in a multivariate logistic regression model. The final model identified increased disease severity assessment grade within 24 hours of admission (OR = 4.12, 95% CI: 2.00–9.35, *P* < .001), elevated preoperative CRP levels (OR = 1.02, 95% CI: 1.01–1.03, *P* < .05), intraoperative blood transfusion (OR = 0.21, 95% CI: 0.10–0.43, *P* < .001), postoperative D-dimer grade > 0.5 (OR = 2.65, 95% CI: 1.08–6.37, *P* < .05), and higher preoperative DVT risk scores (OR = 1.72, 95% CI: 1.35–2.17, *P* < .001) were independent risk factors for postoperative DVT following THA (see Table 2).

**Table 2:** Single-factor analysis.

Variables in the equation							
	B	Std. error	Wald	df	Sig.	Exp(B)	95% CI
Condition assessment within 24 h of admission	1.465	0.393	13.868	1	<.001	4.326	(2–9.35)
Blood transfusion indication (1)	−1.550	0.363	18.198	1	<.001	.212	(0.10–0.43)
DVT risk score	0.537	0.121	19.704	1	<.001	1.711	(1.35–2.17)
D-dimer grading (1)	0.990	0.468	4.479	1	.034	2.691	(1.08–6.37)
Preoperative C-reactive protein	0.018	0.006	8.848	1	.003	1.018	(1.01–1.03)
Quantity	−7.487	1.158	41.800	1	<.001	0.001	

CI = confidence interval, DVT = deep vein thrombosis.

### 4.3. Risk stratification model for deep vein thrombosis after total hip arthroplasty

**Figure 2.**
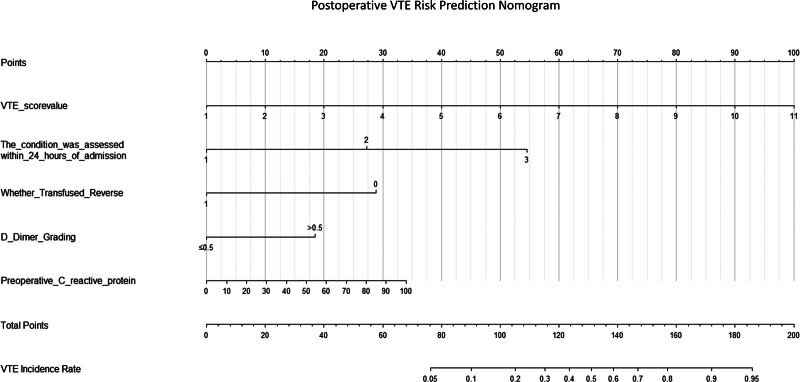
Postoperative VTE risk prediction chart. VTE = venous thromboembolism.

We constructed a nomogram model to predict the risk of developing DVT following THA, based on the 5 independent risk factors described above (Fig. 2). According to the nomogram, the total score is calculated by summing the corresponding scores for each predictor. The predicted probability corresponding to this total score represents the risk of DVT occurrence in THA patients.

### 4.4. Calibration and validation of regression models

**Figure 3.**
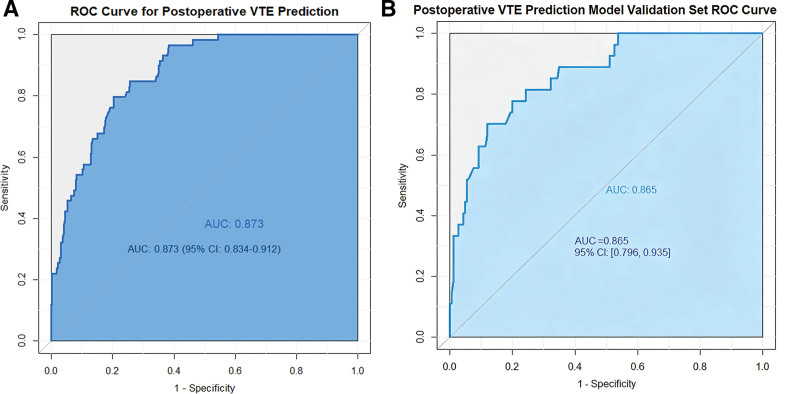
(A) ROC curve. (B) Postoperative VTE prediction model validation set ROC curve. VTE = venous thromboembolism, ROC = receiver operating characteristic.

**Figure 4.**
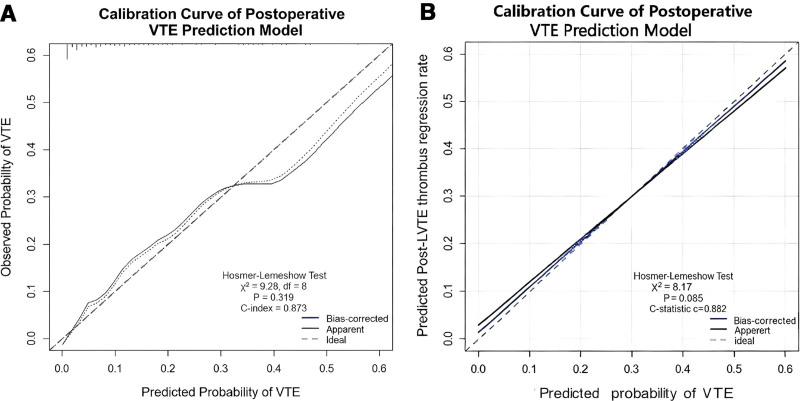
(A) Calibration curve of postoperative VTE prediction model. (B) Calibration curve of post-LVTE thrombus regression model. VTE = venous thromboembolism.

**Figure 5.**
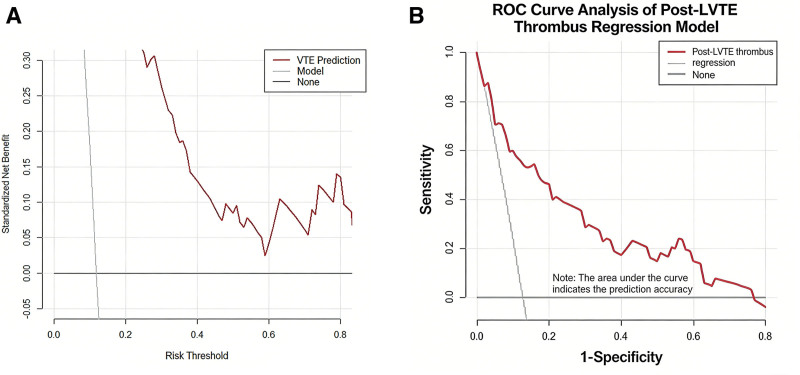
(A) VTE prediction model. (B) Postoperative VTE prediction scoring chart. VTE = venous thromboembolism.

The nomogram developed from the 5 independent predictors exhibited robust discriminatory ability in the training set, with an area under the receiver operating characteristic curve (AUC) of 0.873 (95% CI: 0.832–0.914; see Fig. 3A). After internal validation via 1000 Bootstrap resampling iterations, the model’s optimism-corrected C-index was 0.865. Within the internal validation cohort (DVT incidence: 12.7%, 27/213; see Fig. 3B), the model maintained robust discriminatory performance (AUC = 0.865, 95% CI: 0.798–0.932). Calibration curve analysis (Fig. 4A,B) demonstrated good consistency between the model’s predicted probabilities and the observed DVT incidence rates in both the training and validation cohorts. DCA further confirmed that this predictive model delivers clear net benefit for clinical decision-making across a wide range of threshold probabilities (Fig. 5A,B).

## 5. Discussion

This study demonstrates that a nomogram incorporating readily available preoperative and perioperative variables can effectively predict postoperative DVT risk in elderly patients undergoing hip surgery. The model exhibited excellent discrimination and calibration, and DCA confirmed its clinical net benefit, suggesting its potential utility in individualized risk stratification.

This study identified several independent risk factors for postoperative DVT following hip replacement surgery in elderly patients, including assessment within 24 hours of admission, preoperative CRP levels, history of blood transfusion, D-dimer grading, and DVT risk scoring. Multivariate analysis demonstrated the significant predictive value of these factors, suggesting they may play a key role in the pathogenesis of DVT. Based on these findings, a nomogram prediction model was developed. By integrating scores from each factor, this model enables quantitative assessment of individual patients’ DVT risk, providing a practical tool for early clinical identification of high-risk populations and implementation of stratified interventions.

The results of this study indicate that DVT scores are significantly correlated with the incidence of postoperative DVT in elderly patients undergoing hip surgery, consistent with previous literature findings.^[8]^ Each one-point increase in the Caprini score corresponds to an increased risk of postoperative DVT. However, its applicability to THA patients has certain limitations: Within this scoring system, the items “elective arthroplasty” and “hip, pelvic, or lower extremity fracture” each assign a value of 5 points. When combined with standard age-related scores, the vast majority of THA patients are classified as high-risk (score ≥ 6 points), thereby diminishing its capacity for individualized risk stratification. Existing research has confirmed that independent risk factors for postoperative DVT after THA encompass multiple aspects including age, obesity, metabolic syndrome, cardiovascular disease, history of thrombosis, ASA classification, surgical duration, and blood loss.^[9]^ Despite this, traditional scoring systems remain inadequate for achieving precise prediction of postoperative DVT. In contrast, the nomogram developed in this study addresses this gap by providing a visual, individualized risk estimate based on multivariate regression results. Each predictor’s independent contribution to DVT occurrence is intuitively represented, enabling clinicians to weigh risk factors jointly rather than in isolation. This model demonstrates excellent discrimination and calibration, and DCA reveals clear clinical net benefit. It assists clinicians in individualized risk assessment, enhancing predictive accuracy and clinical utility.

The comprehensive assessment within 24 hours of admission (covering nursing risks, multidisciplinary communication, and feedback) was confirmed as an independent risk factor for postoperative DVT in elderly hip replacement patients (*P* < .05), consistent with previous research findings.^[10]^ Such assessments play a crucial role in DVT prevention by enhancing perioperative management quality. Studies indicate that enhanced pain management promotes early patient mobilization and reduces complication risks^[11]^; while nurse-led rehabilitation interventions help shorten hospital stays and improve functional outcomes.^[12]^ Collectively, these measures alleviate postoperative burdens by optimizing pain control and functional recovery. Furthermore, systematic reviews confirm that interdisciplinary collaboration models significantly improve patient prognosis.^[13]^ Thus, systematic, multidisciplinary early clinical assessment and intervention constitute critical components for reducing postoperative DVT risk.

Preoperative CRP levels were correlated with the occurrence of postoperative DVT. As a well-established biomarker of inflammation triggered by trauma or infection, CRP is also an important indicator of postoperative orthopedic infection.^[14,15]^ Compared to erythrocyte sedimentation rate and white blood cell count, CRP is currently considered a more reliable marker for monitoring inflammation and infectious processes, particularly in the context of surgical complications.^[16,17]^ This study found that preoperative CRP levels were higher in the thrombosis group than in the non-thrombosis group, and elevated CRP emerged as an independent risk factor for postoperative DVT in patients undergoing THA. This conclusion aligns with Ge’s findings,^[18]^ who reported that that this biomarker plays a crucial role in the pathophysiological processes underlying DVT and is valuable for predicting its occurrence.

Previous studies have demonstrated that D-dimer levels are closely associated with the development of postoperative lower extremity DVT. As a fibrin degradation product generated by plasmin-mediated fibrinolysis, D-dimer serves as an essential biomarker of coagulation activation and fibrinolysis activity, and is therefore widely utilized in predicting DVT following fractures.^[19,20]^ Our findings reveal that patients with abnormal D-dimer levels had a significantly higher incidence of postoperative DVT compared to those with normal levels, which is consistent with the results reported by Zhang et al.^[21]^ However, this study revealed that the predictive value of D-dimer for postoperative DVT varied according to the timing of measurement. Specifically, no significant difference in D-dimer levels was observed between the thrombotic and non-thrombotic groups at the time of admission, suggesting that admission D-dimer is not a reliable predictor of postoperative DVT.This lack of association is likely attributable to the fact that all subjects in this study were trauma patients with fractures, and trauma itself is an independent risk factor for elevated D-dimer, thereby diminishing its specificity for predicting thrombotic events.^[22]^ This interpretation is supported by Han et al,^[23]^ who reported a 97.2% positivity rate for D-dimer at admission among elderly patients with hip fractures, indicating that the trauma-induced stress response may mask early thrombotic signals. In contrast, postoperative D-dimer levels were significantly higher in the thrombosis group than in the non-thrombosis group, establishing postoperative D-dimer as an independent risk factor for DVT following THA.^[23]^ This finding strongly aligns with Zhuang’s^[19]^ conclusion that preoperative D-dimer is poorly predictive due to fracture interference, whereas elevated postoperative D-dimer serves as an effective predictor for early DVT diagnosis. Thus, dynamic postoperative monitoring of D-dimer levels holds significant clinical value for early DVT screening in hip fracture patients undergoing arthroplasty.

In contrast to most previous reports, perioperative blood transfusion demonstrated a negative correlation with postoperative DVT incidence in this study, suggesting a potential protective effect. This finding diverges from the prevailing view that components in stored blood – such as cellular debris and microemboli – may impair coagulation and potentially increase DVT risk.^[24,25]^ The observed discrepancy may be attributed to confounding variables: transfusion recipients typically experience more rigorous perioperative hydration and enhanced monitoring,^[26]^ often due to substantial blood loss or preexisting anemia.These enhanced interventions themselves contribute to reduced DVT rates.^[27]^ Thus, transfusion is more likely an indicator of intensive perioperative care rather than a direct causal protective factor.^[28]^

### 5.1. Strengthens

This study has several strengths. First, the nomogram was developed specifically for the elderly hip surgery population, addressing the limited discriminatory capacity of general risk assessment tools in this cohort. Second, the model incorporates routinely available clinical variables, enhancing its applicability in real-world perioperative settings. Finally, internal validation demonstrated robust predictive performance, supporting its potential utility for individualized risk stratification.

### 5.2. Limitations

Our study has several limitations. First, the results are restricted to in-hospital outcomes, as long-term follow-up data beyond discharge were not available. Given that thrombotic events may occur after discharge, particularly following the cessation of routine prophylaxis, the absence of post-discharge surveillance may underestimate the true incidence of DVT. Second, due to constraints of our data system, we were unable to track patients who were transferred to rehabilitation facilities approximately one week post-surgery, limiting our ability to assess outcomes during the subacute recovery phase. Despite these limitations, our findings provide clinically actionable insights for in-hospital DVT risk stratification, offering a practical tool for identifying high-risk patients during the acute postoperative period.

## 6. Conclusion

In conclusion, this study identified key independent risk factors for postoperative DVT in elderly patients undergoing hip replacement surgery and developed a corresponding nomogram prediction model. By enabling early identification of high-risk individuals, the model supports the implementation of targeted preventive interventions in clinical practice. Ultimately, this approach holds promise for improving postoperative recovery outcomes and enhancing long-term quality of life in this vulnerable patient population.

## Author contributions

**Conceptualization:** Zheng Xu, Weiting Wu, Xing Liu, Xuli Yang.

**Data curation:** Zheng Xu, Xing Liu .

**Methodology:** Zheng Xu, Weiting Wu, Jie Liu, Xuli Yang.

**Writing – original draft:** Zheng Xu, Jie Liu.

**Writing – review & editing:** Jie Liu, Xuli Yang.
